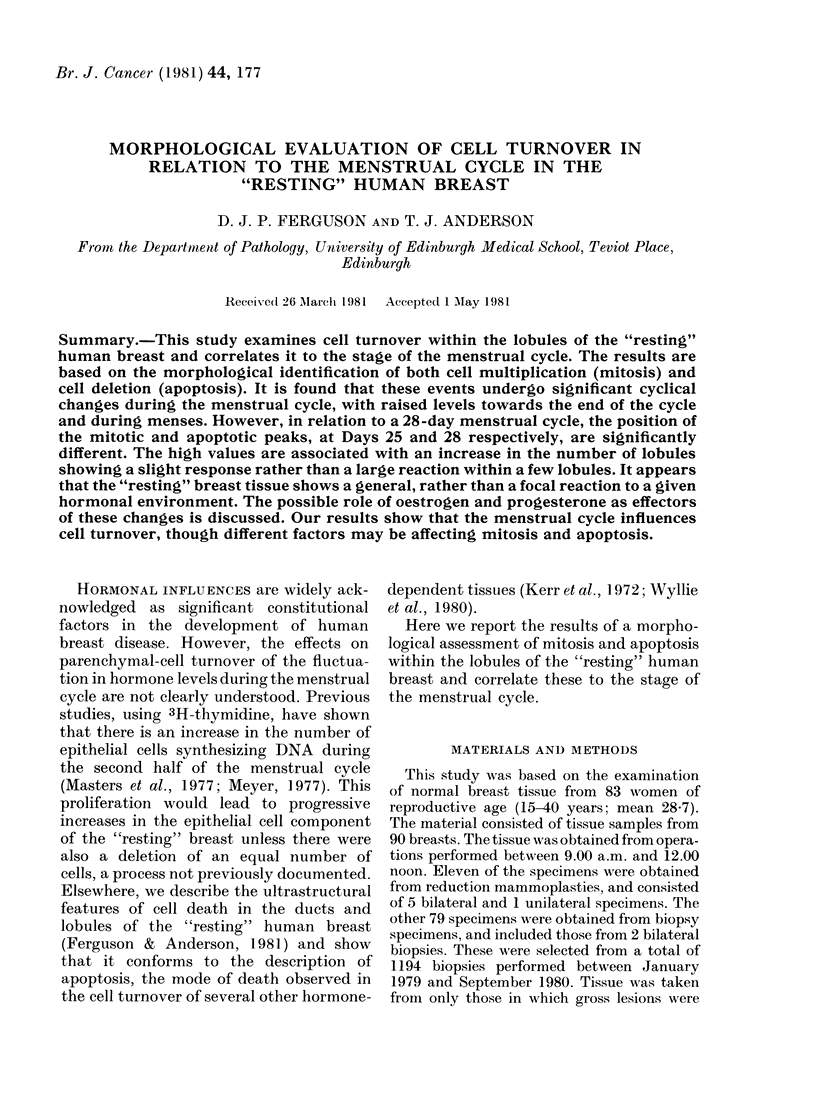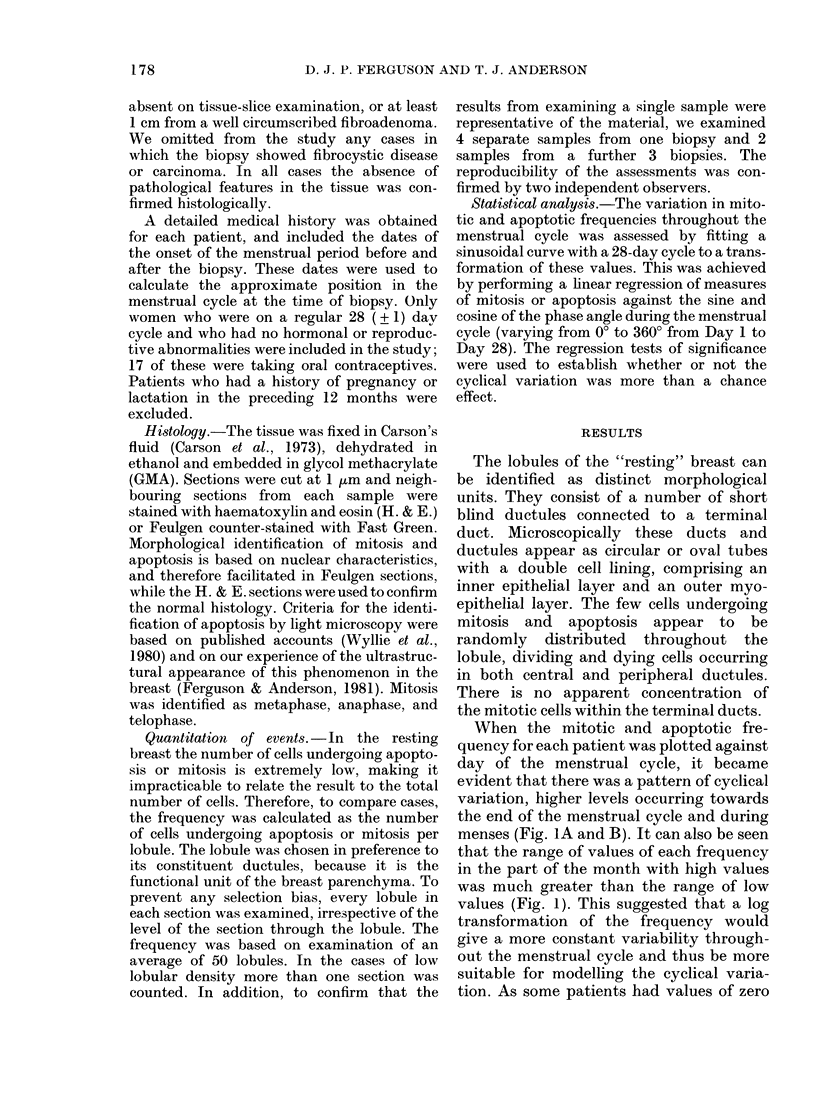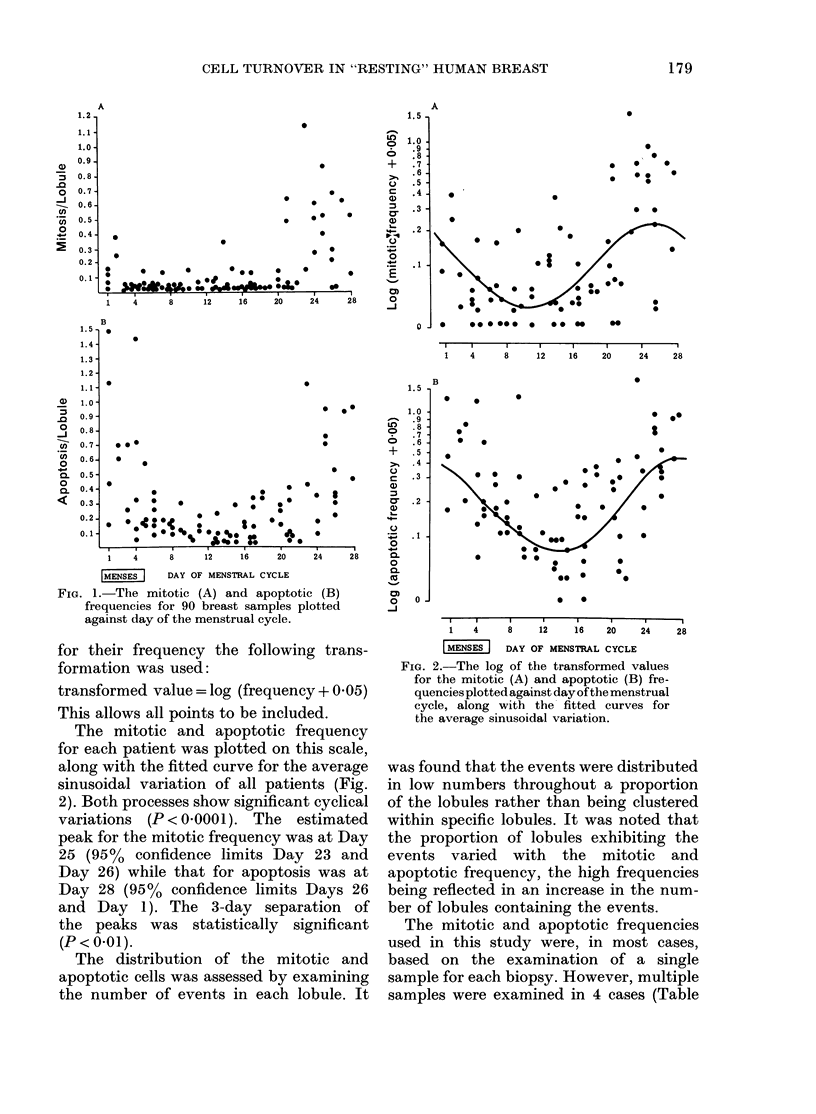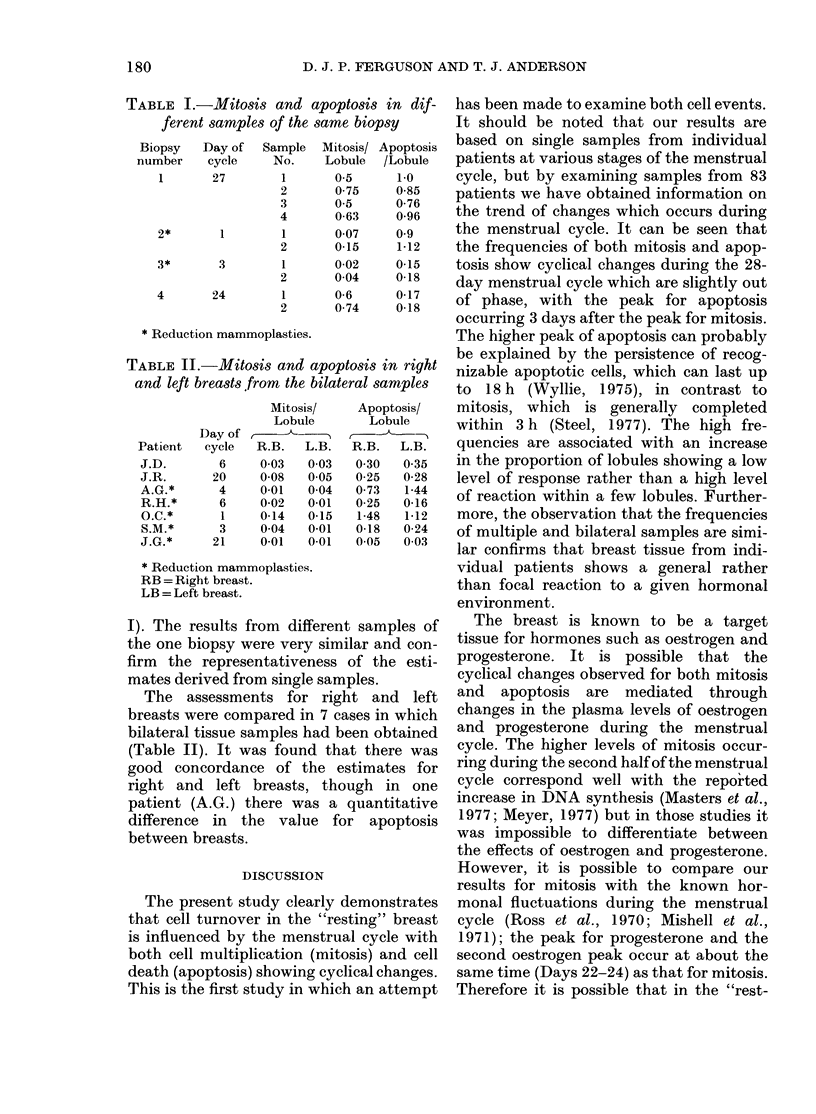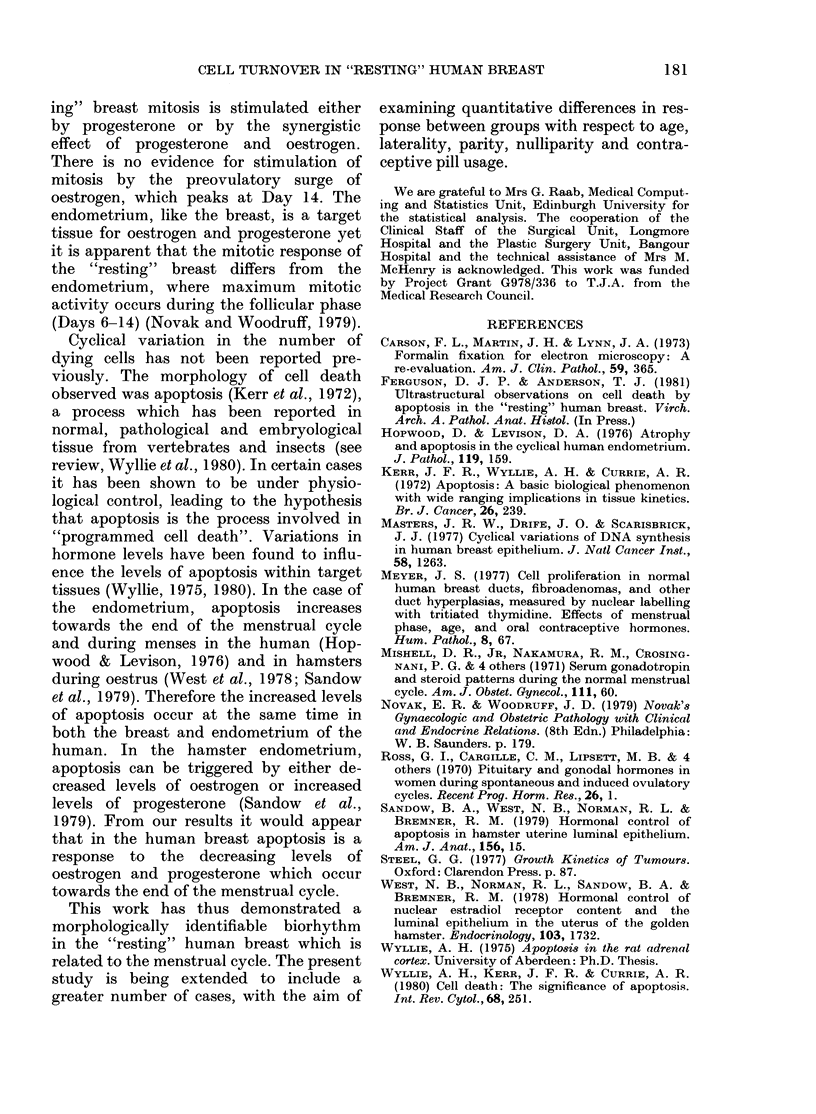# Morphological evaluation of cell turnover in relation to the menstrual cycle in the "resting" human breast.

**DOI:** 10.1038/bjc.1981.168

**Published:** 1981-08

**Authors:** D. J. Ferguson, T. J. Anderson

## Abstract

This study examines cell turnover within the lobules of the "resting" human breast and correlates it to the stage of the menstrual cycle. The results are based on the morphological identification of both cell multiplication (mitosis) and cell deletion (apoptosis). It is found that these events undergo significant cyclical changes during the menstrual cycle, with raised levels towards the end of the cycle and during menses. However, in relation to a 28-day menstrual cycle, the position of the mitotic and apoptotic peaks, at Days 25 and 28 respectively, are significantly different. The high values are associated with an increase in the number of lobules showing a slight response rather than a large reaction within a few lobules. It appears that the "resting" breast tissue shows a general, rather than a focal reaction to a given hormonal environment. The possible role of oestrogen and progesterone as effectors of these changes is discussed. Our results show that the menstrual cycle influences cell turnover, though different factors may be affecting mitosis and apoptosis.


*Br. J. Cancer* (1981) **44,** 177

### MORPHOLOGICAL EVALUATION OF CELL TURNOVER IN RELATION TO THE MENSTRUAL CYCLE IN THE "RESTING" HUMAN BREAST

D. J. P. FERGUSON AND T. J. ANDERSON

*From the Department of Pathology, University of Edinburgh Medical School, Teviot Place, Edinburgh*

Received 26 March 1981 Accepted 1 May 1981

**Summary.—This study examines cell turnover within the lobules of the "resting" human breast and correlates it to the stage of the menstrual cycle. The results are based on the morphological identification of both cell multiplication (mitosis) and cell deletion (apoptosis). It is found that these events undergo significant cyclical changes during the menstrual cycle, with raised levels towards the end of the cycle and during menses. However, in relation to a 28-day menstrual cycle, the position of the mitotic and apoptotic peaks, at Days 25 and 28 respectively, are significantly different. The high values are associated with an increase in the number of lobules showing a slight response rather than a large reaction within a few lobules. It appears that the "resting" breast tissue shows a general, rather than a focal reaction to a given hormonal environment. The possible role of oestrogen and progesterone as effectors of these changes is discussed. Our results show that the menstrual cycle influences cell turnover, though different factors may be affecting mitosis and apoptosis.**

HORMONAL INFLUENCES are widely acknowledged as significant constitutional factors in the development of human breast disease. However, the effects on parenchymal-cell turnover of the fluctuation in hormone levels during the menstrual cycle are not clearly understood. Previous studies, using $^3$H-thymidine, have shown that there is an increase in the number of epithelial cells synthesizing DNA during the second half of the menstrual cycle (Masters *et al.*, 1977; Meyer, 1977). This proliferation would lead to progressive increases in the epithelial cell component of the "resting" breast unless there were also a deletion of an equal number of cells, a process not previously documented. Elsewhere, we describe the ultrastructural features of cell death in the ducts and lobules of the "resting" human breast (Ferguson & Anderson, 1981) and show that it conforms to the description of apoptosis, the mode of death observed in the cell turnover of several other hormone-dependent tissues (Kerr *et al.*, 1972; Wyllie *et al.*, 1980).

Here we report the results of a morphological assessment of mitosis and apoptosis within the lobules of the "resting" human breast and correlate these to the stage of the menstrual cycle.

### MATERIALS AND METHODS

This study was based on the examination of normal breast tissue from 83 women of reproductive age (15–40 years; mean 28·7). The material consisted of tissue samples from 90 breasts. The tissue was obtained from operations performed between 9.00 a.m. and 12.00 noon. Eleven of the specimens were obtained from reduction mammoplasties, and consisted of 5 bilateral and 1 unilateral specimens. The other 79 specimens were obtained from biopsy specimens, and included those from 2 bilateral biopsies. These were selected from a total of 1194 biopsies performed between January 1979 and September 1980. Tissue was taken from only those in which gross lesions were absent on tissue-slice examination, or at least 1 cm from a well circumscribed fibroadenoma. We omitted from the study any cases in which the biopsy showed fibrocystic disease or carcinoma. In all cases the absence of pathological features in the tissue was confirmed histologically.

A detailed medical history was obtained for each patient, and included the dates of the onset of the menstrual period before and after the biopsy. These dates were used to calculate the approximate position in the menstrual cycle at the time of biopsy. Only women who were on a regular 28 ($\pm 1$) day cycle and who had no hormonal or reproductive abnormalities were included in the study; 17 of these were taking oral contraceptives. Patients who had a history of pregnancy or lactation in the preceding 12 months were excluded.

*Histology.*—The tissue was fixed in Carson's fluid (Carson *et al.*, 1973), dehydrated in ethanol and embedded in glycol methacrylate (GMA). Sections were cut at 1 $\mu$m and neighbouring sections from each sample were stained with haematoxylin and eosin (H. & E.) or Feulgen counter-stained with Fast Green. Morphological identification of mitosis and apoptosis is based on nuclear characteristics, and therefore facilitated in Feulgen sections, while the H. & E. sections were used to confirm the normal histology. Criteria for the identification of apoptosis by light microscopy were based on published accounts (Wyllie *et al.*, 1980) and on our experience of the ultrastructural appearance of this phenomenon in the breast (Ferguson & Anderson, 1981). Mitosis was identified as metaphase, anaphase, and telophase.

*Quantitation of events.*—In the resting breast the number of cells undergoing apoptosis or mitosis is extremely low, making it impracticable to relate the result to the total number of cells. Therefore, to compare cases, the frequency was calculated as the number of cells undergoing apoptosis or mitosis per lobule. The lobule was chosen in preference to its constituent ductules, because it is the functional unit of the breast parenchyma. To prevent any selection bias, every lobule in each section was examined, irrespective of the level of the section through the lobule. The frequency was based on examination of an average of 50 lobules. In the cases of low lobular density more than one section was counted. In addition, to confirm that the results from examining a single sample were representative of the material, we examined 4 separate samples from one biopsy and 2 samples from a further 3 biopsies. The reproducibility of the assessments was confirmed by two independent observers.

*Statistical analysis.*—The variation in mitotic and apoptotic frequencies throughout the menstrual cycle was assessed by fitting a sinusoidal curve with a 28-day cycle to a transformation of these values. This was achieved by performing a linear regression of measures of mitosis or apoptosis against the sine and cosine of the phase angle during the menstrual cycle (varying from 0° to 360° from Day 1 to Day 28). The regression tests of significance were used to establish whether or not the cyclical variation was more than a chance effect.

### RESULTS

The lobules of the "resting" breast can be identified as distinct morphological units. They consist of a number of short blind ductules connected to a terminal duct. Microscopically these ducts and ductules appear as circular or oval tubes with a double cell lining, comprising an inner epithelial layer and an outer myoepithelial layer. The few cells undergoing mitosis and apoptosis appear to be randomly distributed throughout the lobule, dividing and dying cells occurring in both central and peripheral ductules. There is no apparent concentration of the mitotic cells within the terminal ducts.

When the mitotic and apoptotic frequency for each patient was plotted against day of the menstrual cycle, it became evident that there was a pattern of cyclical variation, higher levels occurring towards the end of the menstrual cycle and during menses (Fig. 1A and B). It can also be seen that the range of values of each frequency in the part of the month with high values was much greater than the range of low values (Fig. 1). This suggested that a log transformation of the frequency would give a more constant variability throughout the menstrual cycle and thus be more suitable for modelling the cyclical variation. As some patients had values of zero

FIG. 1.—The mitotic (A) and apoptotic (B) frequencies for 90 breast samples plotted against day of the menstrual cycle.

FIG. 2.—The log of the transformed values for the mitotic (A) and apoptotic (B) frequencies plotted against day of the menstrual cycle, along with the fitted curves for the average sinusoidal variation.

for their frequency the following transformation was used:

transformed value = log (frequency + 0·05)

This allows all points to be included.

The mitotic and apoptotic frequency for each patient was plotted on this scale, along with the fitted curve for the average sinusoidal variation of all patients (Fig. 2). Both processes show significant cyclical variations ($P < 0{\cdot}0001$). The estimated peak for the mitotic frequency was at Day 25 (95% confidence limits Day 23 and Day 26) while that for apoptosis was at Day 28 (95% confidence limits Days 26 and Day 1). The 3-day separation of the peaks was statistically significant ($P < 0{\cdot}01$).

The distribution of the mitotic and apoptotic cells was assessed by examining the number of events in each lobule. It was found that the events were distributed in low numbers throughout a proportion of the lobules rather than being clustered within specific lobules. It was noted that the proportion of lobules exhibiting the events varied with the mitotic and apoptotic frequency, the high frequencies being reflected in an increase in the number of lobules containing the events.

The mitotic and apoptotic frequencies used in this study were, in most cases, based on the examination of a single sample for each biopsy. However, multiple samples were examined in 4 cases (Table

TABLE I.—*Mitosis and apoptosis in different samples of the same biopsy*

| Biopsy number | Day of cycle | Sample No. | Mitosis/ Lobule | Apoptosis /Lobule |
|---|---|---|---|---|
| 1 | 27 | 1 | 0·5 | 1·0 |
| | | 2 | 0·75 | 0·85 |
| | | 3 | 0·5 | 0·76 |
| | | 4 | 0·63 | 0·96 |
| 2* | 1 | 1 | 0·07 | 0·9 |
| | | 2 | 0·15 | 1·12 |
| 3* | 3 | 1 | 0·02 | 0·15 |
| | | 2 | 0·04 | 0·18 |
| 4 | 24 | 1 | 0·6 | 0·17 |
| | | 2 | 0·74 | 0·18 |

* Reduction mammoplasties.

TABLE II.—*Mitosis and apoptosis in right and left breasts from the bilateral samples*

| Patient | Day of cycle | Mitosis/ Lobule | | Apoptosis/ Lobule | |
|---|---|---|---|---|---|
| | | R.B. | L.B. | R.B. | L.B. |
| J.D. | 6 | 0·03 | 0·03 | 0·30 | 0·35 |
| J.R. | 20 | 0·08 | 0·05 | 0·25 | 0·28 |
| A.G.* | 4 | 0·01 | 0·04 | 0·73 | 1·44 |
| R.H.* | 6 | 0·02 | 0·01 | 0·25 | 0·16 |
| O.C.* | 1 | 0·14 | 0·15 | 1·48 | 1·12 |
| S.M.* | 3 | 0·04 | 0·01 | 0·18 | 0·24 |
| J.G.* | 21 | 0·01 | 0·01 | 0·05 | 0·03 |

* Reduction mammoplasties.
RB = Right breast.
LB = Left breast.

I). The results from different samples of the one biopsy were very similar and confirm the representativeness of the estimates derived from single samples.

The assessments for right and left breasts were compared in 7 cases in which bilateral tissue samples had been obtained (Table II). It was found that there was good concordance of the estimates for right and left breasts, though in one patient (A.G.) there was a quantitative difference in the value for apoptosis between breasts.

### DISCUSSION

The present study clearly demonstrates that cell turnover in the "resting" breast is influenced by the menstrual cycle with both cell multiplication (mitosis) and cell death (apoptosis) showing cyclical changes. This is the first study in which an attempt has been made to examine both cell events. It should be noted that our results are based on single samples from individual patients at various stages of the menstrual cycle, but by examining samples from 83 patients we have obtained information on the trend of changes which occurs during the menstrual cycle. It can be seen that the frequencies of both mitosis and apoptosis show cyclical changes during the 28-day menstrual cycle which are slightly out of phase, with the peak for apoptosis occurring 3 days after the peak for mitosis. The higher peak of apoptosis can probably be explained by the persistence of recognizable apoptotic cells, which can last up to 18 h (Wyllie, 1975), in contrast to mitosis, which is generally completed within 3 h (Steel, 1977). The high frequencies are associated with an increase in the proportion of lobules showing a low level of response rather than a high level of reaction within a few lobules. Furthermore, the observation that the frequencies of multiple and bilateral samples are similar confirms that breast tissue from individual patients shows a general rather than focal reaction to a given hormonal environment.

The breast is known to be a target tissue for hormones such as oestrogen and progesterone. It is possible that the cyclical changes observed for both mitosis and apoptosis are mediated through changes in the plasma levels of oestrogen and progesterone during the menstrual cycle. The higher levels of mitosis occurring during the second half of the menstrual cycle correspond well with the reported increase in DNA synthesis (Masters *et al.*, 1977; Meyer, 1977) but in those studies it was impossible to differentiate between the effects of oestrogen and progesterone. However, it is possible to compare our results for mitosis with the known hormonal fluctuations during the menstrual cycle (Ross *et al.*, 1970; Mishell *et al.*, 1971); the peak for progesterone and the second oestrogen peak occur at about the same time (Days 22–24) as that for mitosis. Therefore it is possible that in the "resting" breast mitosis is stimulated either by progesterone or by the synergistic effect of progesterone and oestrogen. There is no evidence for stimulation of mitosis by the preovulatory surge of oestrogen, which peaks at Day 14. The endometrium, like the breast, is a target tissue for oestrogen and progesterone yet it is apparent that the mitotic response of the "resting" breast differs from the endometrium, where maximum mitotic activity occurs during the follicular phase (Days 6–14) (Novak and Woodruff, 1979).

Cyclical variation in the number of dying cells has not been reported previously. The morphology of cell death observed was apoptosis (Kerr *et al.*, 1972), a process which has been reported in normal, pathological and embryological tissue from vertebrates and insects (see review, Wyllie *et al.*, 1980). In certain cases it has been shown to be under physiological control, leading to the hypothesis that apoptosis is the process involved in "programmed cell death". Variations in hormone levels have been found to influence the levels of apoptosis within target tissues (Wyllie, 1975, 1980). In the case of the endometrium, apoptosis increases towards the end of the menstrual cycle and during menses in the human (Hopwood & Levison, 1976) and in hamsters during oestrus (West *et al.*, 1978; Sandow *et al.*, 1979). Therefore the increased levels of apoptosis occur at the same time in both the breast and endometrium of the human. In the hamster endometrium, apoptosis can be triggered by either decreased levels of oestrogen or increased levels of progesterone (Sandow *et al.*, 1979). From our results it would appear that in the human breast apoptosis is a response to the decreasing levels of oestrogen and progesterone which occur towards the end of the menstrual cycle.

This work has thus demonstrated a morphologically identifiable biorhythm in the "resting" human breast which is related to the menstrual cycle. The present study is being extended to include a greater number of cases, with the aim of examining quantitative differences in response between groups with respect to age, laterality, parity, nulliparity and contraceptive pill usage.

We are grateful to Mrs G. Raab, Medical Computing and Statistics Unit, Edinburgh University for the statistical analysis. The cooperation of the Clinical Staff of the Surgical Unit, Longmore Hospital and the Plastic Surgery Unit, Bangour Hospital and the technical assistance of Mrs M. McHenry is acknowledged. This work was funded by Project Grant G978/336 to T.J.A. from the Medical Research Council.

### REFERENCES

CARSON, F. L., MARTIN, J. H. & LYNN, J. A. (1973) Formalin fixation for electron microscopy: A re-evaluation. *Am. J. Clin. Pathol.*, **59**, 365.

FERGUSON, D. J. P. & ANDERSON, T. J. (1981) Ultrastructural observations on cell death by apoptosis in the "resting" human breast. *Virch. Arch. A. Pathol. Anat. Histol.* (In Press.)

HOPWOOD, D. & LEVISON, D. A. (1976) Atrophy and apoptosis in the cyclical human endometrium. *J. Pathol.*, **119**, 159.

KERR, J. F. R., WYLLIE, A. H. & CURRIE, A. R. (1972) Apoptosis: A basic biological phenomenon with wide ranging implications in tissue kinetics. *Br. J. Cancer*, **26**, 239.

MASTERS, J. R. W., DRIFE, J. O. & SCARISBRICK, J. J. (1977) Cyclical variations of DNA synthesis in human breast epithelium. *J. Natl Cancer Inst.*, **58**, 1263.

MEYER, J. S. (1977) Cell proliferation in normal human breast ducts, fibroadenomas, and other duct hyperplasias, measured by nuclear labelling with tritiated thymidine. Effects of menstrual phase, age, and oral contraceptive hormones. *Hum. Pathol.*, **8**, 67.

MISHELL, D. R., JR, NAKAMURA, R. M., CROSINGNANI, P. G. & 4 others (1971) Serum gonadotropin and steroid patterns during the normal menstrual cycle. *Am. J. Obstet. Gynecol.*, **111**, 60.

NOVAK, E. R. & WOODRUFF, J. D. (1979) *Novak's Gynaecologic and Obstetric Pathology with Clinical and Endocrine Relations.* (8th Edn.) Philadelphia: W. B. Saunders. p. 179.

ROSS, G. I., CARGILLE, C. M., LIPSETT, M. B. & 4 others (1970) Pituitary and gonodal hormones in women during spontaneous and induced ovulatory cycles. *Recent Prog. Horm. Res.*, **26**, 1.

SANDOW, B. A., WEST, N. B., NORMAN, R. L. & BREMNER, R. M. (1979) Hormonal control of apoptosis in hamster uterine luminal epithelium. *Am. J. Anat.*, **156**, 15.

STEEL, G. G. (1977) *Growth Kinetics of Tumours.* Oxford: Clarendon Press. p. 87.

WEST, N. B., NORMAN, R. L., SANDOW, B. A. & BREMNER, R. M. (1978) Hormonal control of nuclear estradiol receptor content and the luminal epithelium in the uterus of the golden hamster. *Endocrinology*, **103**, 1732.

WYLLIE, A. H. (1975) *Apoptosis in the rat adrenal cortex.* University of Aberdeen: Ph.D. Thesis.

WYLLIE, A. H., KERR, J. F. R. & CURRIE, A. R. (1980) Cell death: The significance of apoptosis. *Int. Rev. Cytol.*, **68**, 251.